# Controlled Release of Thymol from Poly(Lactic Acid)-Based Silver Nanocomposite Films with Antibacterial and Antioxidant Activity

**DOI:** 10.3390/antiox9050395

**Published:** 2020-05-07

**Authors:** Marina Ramos, Ana Beltran, Elena Fortunati, Mercedes Peltzer, Francesco Cristofaro, Livia Visai, Artur J.M. Valente, Alfonso Jiménez, José María Kenny, María Carmen Garrigós

**Affiliations:** 1 Department of Analytical Chemistry, Nutrition & Food Sciences, University of Alicante, 03080 Alicante, Spain; ana.beltran@ua.es (A.B.); alfjimenez@ua.es (A.J.); mc.garrigos@ua.es (M.C.G.); 2 Civil Environmental Engineering Department, University of Perugia, UdR INSTM, Strada di Pentima 4, 05100 Terni, Italy; elenafortunati@gmail.com (E.F.); jose.kenny@unipg.it (J.M.K.); 3 Departamento de Ciencia y Tecnología, Universidad Nacional de Quilmes, Bernal, Buenos Aires B1876BXD, Argentina; mercedes.peltzer@unq.edu.ar; 4 Consejo Nacional de Investigaciones Científicas y Técnicas (CONICET), Ciudad Autónoma de Buenos Aires (CABA) C1425FQB, Argentina; 5 Department of Molecular Medicine, Center for Health Technologies (C.H.T.), UdR INSTM, University of Pavia, 27100 Pavia, Italy; francesco.cristofaro01@universitadipavia.it (F.C.); livia.visai@unipv.it (L.V.); 6 Department of Occupational Medicine, Toxicology and Environmental Risks, Istituti Clinici Scientifici (ICS) Maugeri, Società Benefit S.p.A IRCCS, 27100 Pavia, Italy; 7 Department of Chemistry, University of Coimbra, CQC, 3004-535 Coimbra, Portugal; avalente@ci.uc.pt

**Keywords:** active packaging, kinetic release, thymol, silver nanoparticles, antioxidant capacity, poly(lactic acid), antibacterial

## Abstract

Thymol and silver nanoparticles (Ag-NPs) were used to develop poly(lactic acid) (PLA)-based films with antioxidant and antibacterial performance. Different amounts of thymol (6 and 8 wt%) and 1 wt% Ag-NPs were added to PLA to produce the active films. Ag-NPs and thymol were successfully identified in the nanocomposite structures using spectroscopic techniques. A kinetic study was performed to evaluate the release of thymol and Ag-NPs from the nanocomposites to an aqueous food simulant (ethanol 10%, v/v) at 40 °C. The diffusion of thymol from the polymer matrix was affected by the presence of non-migrating Ag-NPs, which showed non-Fickian release behavior. The ternary system including 1 wt% Ag-NPs and 8 wt% thymol showed clear antibacterial performance by reducing the cell viability of *Escherichia coli* and *Staphylococcus aureus* by around 40% after 3 and 24 h of storage at 4, 25, and 37 °C compared to neat PLA. Significant antioxidant behavior of all active films was also confirmed using the 2,2-diphenyl-1-picrylhydrazyl (DPPH) method. The obtained nanocomposite films based on PLA and the addition of Ag-NPs and thymol were proven to have combined antioxidant and antibacterial performance, with controlled release of thymol. These formulations have potential applications in the development of innovative and customized active packaging systems to increase the shelf-life of food products.

## 1. Introduction

The rising trend of the industrial use of environmentally friendly materials and stringent regulations have been the driving forces behind public demand for the of use of biopolymers. Materials such as poly(lactic acid) (PLA), poly(hydroxyl alkanoates) (PHA), polyethylene furanoate (PEF), thermoplastic starch (TPS), and biodegradable polyesters are interesting alternatives to polymers derived from fossil fuels. This is due to their renewable origin, biodegradability, and biocompatibility, making them a potential solution for the environmental problems caused by the accumulation of petrochemical-based plastic waste [1]. Among them, PLA is one of the most common biopolymers. Films made from PLA show a good balance of performance properties, which have food packaging applications (high mechanical strength, flavor and aroma resistance, high clarity and printability, etc.). Unfortunately, PLA also has some limitations, such as low melt drawability, high permeability to small molecules (oxygen and water), and brittleness [2]. Several strategies have been developed to improve the structural and functional properties of PLA-based materials, including the incorporation of low amounts of nanoparticles (NPs) without relevant alteration of their migration behavior to ensure their suitability for food packaging applications [3]. In terms of innovative packaging concepts, the use of metallic-based NPs instead of antimicrobial organic agents offers some advantages, such as their high antimicrobial efficiency, lack of negative impacts on food sensory properties, and compatibility with harsh polymer processing conditions, making NPs suitable for food spoilage control [4,5,6,7,8]. Tanase et al. demonstrated that synthesized Ag-NPs exhibited antioxidant activity and antibacterial effect against different microbial agents [9]. For example, ZnO-NPs were incorporated into PLA coatings for antimicrobial packaging applications, showing that their incorporation gave the material surface antimicrobial properties against *Escherichia coli* (*E. coli*) and *Staphylococcus aureus* (*S. aureus*) [4]. In addition, Fortunati et al. developed and characterized ternary nano-biocomposite films based on PLA with modified cellulose nanocrystals (s-CNC) and Ag-NPs with high antimicrobial activity against *E. coli* and *S. aureus*. [10]. These films also showed homogeneous Ag dispersion in the polymer matrix, which did not affect the original PLA transparency but showed a clear improvement in barrier properties (40%–45%). Regarding the safety and environmental effects of the products containing Ag-NPs in direct contact with food, recent studies have reported that these types of substances can be used for food preservation, considering their quality and safety, which are desirable in food technology [11]. In some works, the migration levels are clearly below the legislative migration limits in Europe set by the EU Regulation No. 10/2011 (for plastic materials and articles intended to come into contact with food), such as in poly(vinyl chloride) (PVC) and PLA nanocomposites [12,13,14].

Current trends in food science focus on improving the food quality, safety, and shelf-life, while maintaining natural properties and conditions. Therefore, the search for successful combinations of natural additives with intrinsic antioxidant properties and nanoparticles has resulted in some recent research on active nano-biocomposites [15,16,17]. Thymol is one of the major compounds present in thyme and oregano essential oils, which has demonstrated high antimicrobial activity [18] and antioxidant performance [19,20,21,22]. The use of a combination of thymol and Ag-NPs to obtain active nanocomposites with both antioxidant and antimicrobial properties has not been extensively studied and it represents an interesting approach to reducing the oxidative and microbial deterioration of food products in order to increase their quality and shelf-life. Therefore, the proposed work could help to fill in the gaps in the development of new sustainable nanocomposite films with combined antioxidant and antibacterial performance, while maintaining low Ag-NP migration and controlled thymol release, in order to validate the potential use of these materials in active packaging applications.

In this work, nanocomposite films based on PLA, thymol, and Ag-NPs were developed and characterized in terms of antioxidant and antibacterial performance against *E. coli* and *S. aureus* bacteria. The added additives were identified using spectroscopic techniques. The release of thymol and Ag-NPs to an aqueous food simulant (ethanol 10%, v/v) at 40 °C was also evaluated using kinetic models. Analytical methods based on high-performance liquid chromatography–ultraviolet detection (HPLC-UV) and inductively coupled plasma–mass spectrometry (ICP-MS) were optimized and validated for such purpose.

## 2. Materials and Methods

### 2.1. Materials and Chemical Reagents

Commercial poly(lactic acid) PLA-4060D (T_g_ = 58 °C, 11–13 wt% D-isomer) was supplied in pellets by NatureWorks Co., (Minnetonka, MN, USA). Methanol (MeOH, HPLC grade), ethanol (EtOH, HPLC grade), acetonitrile (ACN, HPLC grade), 2,2-diphenyl-1-picrylhydrazyl (DPPH, 95%), and thymol (99.5%) were purchased from Sigma-Aldrich (Madrid, Spain). Commercial Ag-NP P203, with a size distribution range between 20 and 80 nm, was purchased from Cima Nano-Tech (Saint Paul, MN, USA) and was previously treated at 700 °C for 1 h to condition the nanomaterial for further processing, as reported elsewhere [23].

### 2.2. Active Nano-Biocomposite Preparation

PLA pellets were dried overnight at 45 °C before extrusion to prevent polymer hydrolysis during processing [24]. Different binary and ternary PLA-based films with thymol and Ag-NPs were obtained by using a twin-screw microextruder (DSM Explore 5 & 15 CC Micro Compounder, Heerlen, The Netherlands) [25]. Ag-NPs (1 wt%) were added directly to the melted PLA matrix. Thymol was added in the last 3 min of the extrusion process and the screw speed was then reduced to 100 rpm to limit losses by vaporization and thymol decomposition by high temperatures and shear stress. The thickness of the developed films was determined at five random positions using a 293 MDC-Lite Digimatic Micrometer (Mitutoyo, Japan), obtaining average thickness values of around 40 µm.

Table 1 summarizes the composition and some properties of the binary and ternary systems, containing different amounts of thymol (6 wt% and 8 wt%) or 1 wt% Ag-NPs with respect to the polymer content. PLA without any additive was also prepared as a control sample. The optical and morphological properties of the developed films were reported in a previous study [25].

### 2.3. Identification of Thymol and Ag-NPs in PLA-Based Films

Thymol was identified in PLA matrices by attenuated total reflectance–Fourier transform infrared spectroscopy (ATR-FTIR), using a Jasco FTIR 615 spectrometer (Easton, MD, USA) equipped with a deuterated triglycine sulfate (DTGS) detector. Spectra were recorded in the absorbance mode in the 4000–400 cm^−1^ range, using 64 scans and 4 cm^−1^ resolution, which were corrected against a background spectrum of air. Two spectra replicates were obtained for each sample.

UV-VIS spectroscopy was used to detect the characteristic bands of Ag-NPs and thymol. A Perkin Elmer Instruments (Lambda 35) UV-VIS spectrophotometer (Waltham, MA, USA) operating in the 250–500 nm range was used.

X-ray diffraction (XRD) patterns were recorded at room temperature at scattering angles (2θ) ranging from 2.5° to 80° (step size = 0.05° min^−1^) using filtered Cu Kα radiation (λ = 1.54 Å). A Bruker D8-Advance diffractometer (Madison, WI, USA) was used, with X-ray tube voltage and current of 40 kV and 40 mA, respectively.

### 2.4. Quantification of Thymol in PLA-Based Films after Processing

The amount of thymol present in the PLA-based films after processing was determined by solid–liquid extraction followed by HPLC-UV analysis. Then, 0.05 ± 0.01 g of each film was extracted with 10 mL of methanol at 40 °C and 50% relative humidity (RH) for 24 h in a climate chamber (Dycometal CM-081, Barcelona, Spain). Three replicates were carried out for each formulation. A Shimadzu LC-20A liquid chromatograph (Kyoto, Japan) with a UV detector and a LiChrospher 100 RP18 column (250 mm × 5 mm × 5 μm, Agilent Technologies, USA) was used. An isocratic elution of 40:60 (v:v) acetonitrile/water at 25 °C and a flow rate of 1 mL min^−1^ was applied. Then, 20 μL of the extracted samples were injected and analyses were performed in triplicate at 274 nm. Standard solutions of thymol in methanol at concentrations between 100 and 500 mg kg^−1^ were used to elaborate the calibration curve for the thymol quantification.

### 2.5. Kinetic Release Study of Thymol and Ag-NPs from PLA-Based Films

The release of thymol and Ag-NPs from PLA-based nanocomposite films was evaluated by using ethanol 10% (v/v) as a food simulant, in agreement with the European Standard EN 13130-2005 and the Commission Regulation (EU) No. 10/2011 on plastic materials and articles intended to come into contact with food [12]. Total immersion migration tests were performed with 12 cm^2^ films and 20 mL of the simulant (area-to-volume ratio of 6 dm^2^ L^−1^) at 40 °C in an oven (J.P. Selecta, Barcelona, Spain) for 15 days. A blank test was also carried out. Extracts were taken at different times (2, 4, 6, 12, 24, and 48 h; and 5, 10, and 15 days) and were stored at −4 °C before analysis. The amount of thymol and Ag-NPs released from the PLA-based films to the food simulant was determined by HPLC-UV and ICP-MS, respectively. Tests were performed in triplicate.

The amount of Ag-NPs released from nanocomposite films into ethanol 10% (v/v) at different times was directly determined by using an Agilent 7700x inductively coupled plasma–mass spectrometry (ICP-MS) (Santa Clara, CA, USA) and following those conditions reported by Song et al., with some modifications in the experimental parameters [26]. A Scott-type spray chamber (Agilent Technologies) was used for sample introduction connected to a MicroMist small volume nebulizer. The sampling depth was 8.0 mm and argon was used as the carrier gas. ICP-MS operating conditions were: radio frequency (RF) power, 1500 W; plasma gas flow rate, 15.0 L min^−1^; auxiliary gas flow rate, 0.9 L min^−1^; carrier gas flow rate, 1.0 L min^−1^; and make-up gas flow rate, 0.56 L min^−1^. Rhodium was used as internal standard and it was introduced by using a peristaltic pump in line with the sample solution. Suspensions were sonicated for 1 min prior to analysis. Calibration standards were obtained by dilution of a stock solution (0.1 mg kg^−1^) of Ag-NPs in ethanol 10% (v/v) to avoid matrix effects. Dilutions were prepared by accurately weighing the corresponding aliquot of the stock solution after 1 min sonication (±0.1 mg).

The amount of thymol released into ethanol 10% (v/v) was determined in triplicate with an Agilent 1260 Infinity HPLC Diode Array Detector (DAD) (Agilent, Santa Clara, CA, USA) and an Agilent Eclipse Plus C18 (100 mm × 4.6 mm × 3.5 μm) column. The mobile phase was acetonitrile/water (40:60) at 1 mL min^−1^ flow rate. Then, 20 μL of the extracted samples was injected and detection was performed at λ = 274 nm. Different standards between 5 and 500 mg kg^−1^ and working solutions of thymol were prepared in ethanol 10% (v/v).

The analytical figures of merit of the analytical methods were determined. Quantitative parameters such as working ranges, correlation coefficients, limits of detection (LOD), and limits of quantification (LOQ) were obtained to validate the proposed HPLC-UV and ICP-MS methods. LOD and LOQ values were calculated from the regression parameters obtained from the calibration curves (3 S_y/x_/a and 10 S_y/x_/a, respectively; where S_y/x_ is the standard deviation of the residues and a is the slope).

### 2.6. Evaluation of the Antioxidant Activity of the Active PLA-Based Films

The antioxidant activity of the active PLA-based films was evaluated in terms of the radical scavenging ability of the thymol released into ethanol 10% (v/v) by using the DPPH method, as proposed by Byun et al., with slight modifications [27]. Then, 100 μL aliquots of each extract were mixed with 3.9 mL of a methanolic solution of DPPH (23 mg L^−1^) in a capped cuvette. The mixture was shaken vigorously and it was kept in a dark at room temperature for 200 min. The absorbance of each solution was determined at 517 nm using a Biomate-3 UV-VIS spectrophotometer (Thermo Scientific, Madison, USA). All analyses were performed in triplicate and the antioxidant capacity was expressed as the ability to scavenge the stable radical DPPH using Equation (1):

(1) $$DPPH\;scavenging\;activity\;\left(\%\right)=\frac{A_{control}-A_{sample}}{A_{control}}\times 100$$

where *A_Control_* and *A_Sample_* are the absorbances of the blank control at t = 0 min and the tested sample at t = 200 min, respectively.

### 2.7. Determination of the Antibacterial Activity of the Active PLA-Based Films

The microorganisms used in this study were *Escherichia coli RB (E. coli RB)* and *Staphylococcus aureus 8325-4 (S. aureus 8325-4*). *E. coli RB* was an isolate strain provided by the “Zooprofilattico Institute of Pavia” (Italy), whereas *S. aureus 8325-4* was kindly provided by Dr. Timothy J. Foster (Department of Microbiology, Dublin, Ireland). *E. coli RB* and *S. aureus 8325-4* were routinely grown overnight in Luria–Bertani broth (LB) and brain–heart infusion (BHI) (Difco Laboratories Inc., Detroit, MI, USA), respectively, under aerobic conditions at 37 °C using a shaker incubator (New Brunswick Scientific Co., Edison, NJ, USA). These cultures were reduced at a final density of 1 × 10^10^ cells mL^−1^, as determined by comparing the optical density at 600 nm (OD_600_) of samples with a standard curve relating OD_600_ to cell number.

The evaluation of the antibacterial activity of neat PLA and PLA active nanocomposite films was carried out in 100 µL of overnight-diluted cell suspensions (1 × 10^4^ UFC mL^−1^) of *E. coli RB* or *S. aureus 8325-4*. These suspensions were added to each sample, seeded at the bottom of a 96-well tissue culture plate, and further incubated at three different temperatures: 4, 24, and 37 °C for 3 h and 24 h, respectively. Furthermore, 96-well flat-bottom sterile polystyrene culture plates (TCP) used as controls were incubated under the same conditions. At the end of each incubation time, the bacterial suspension was then serially diluted and plated on the Luria–Bertani (LB) (*E. coli RB*) or brain–heart infusion (BHI) (*S. aureus 8325-4*) agar plates, respectively. Plates were then incubated for 24 or 48 h at 37 °C. Cell survival was expressed as the percentage of CFU of bacterial growth on PLA active nanocomposite films compared to that obtained for the neat PLA film.

### 2.8. Statistical Analysis

Statistical analysis of the results was performed with the SPSS commercial software (Version 15.0, Chicago, IL, USA). A one-way analysis of variance (ANOVA) was carried out. Differences between mean values were assessed on the basis of confidence intervals using Tukey’s test at a *p* < 0.05 significance level.

Two-group comparisons were performed by application of the Student’s *t*-test for the antibacterial activity, and results were expressed as mean ± SD (standard deviation) using GraphPad Prism 4.0 software (San Diego, CA, USA). Two-tailed *p* values < 0.05 were considered statistically significant.

## 3. Results

### 3.1. Identification of Thymol and Ag-NPs in PLA-Based Films

**Figure 1:**
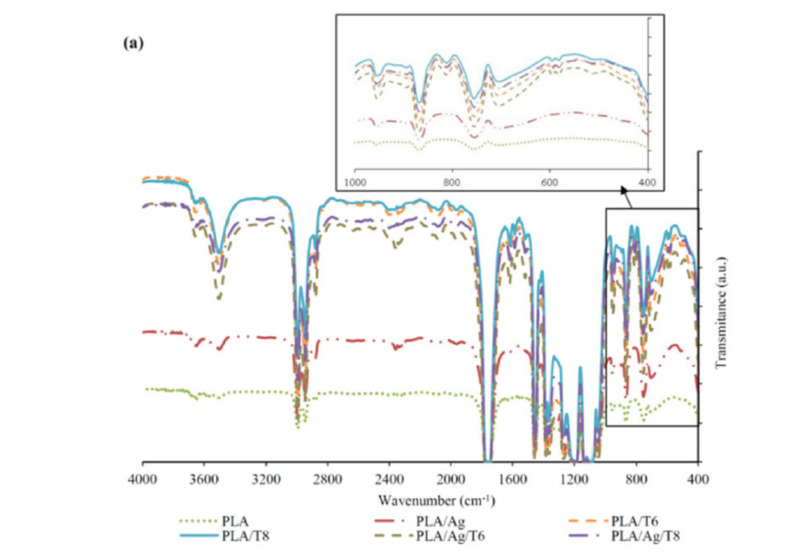

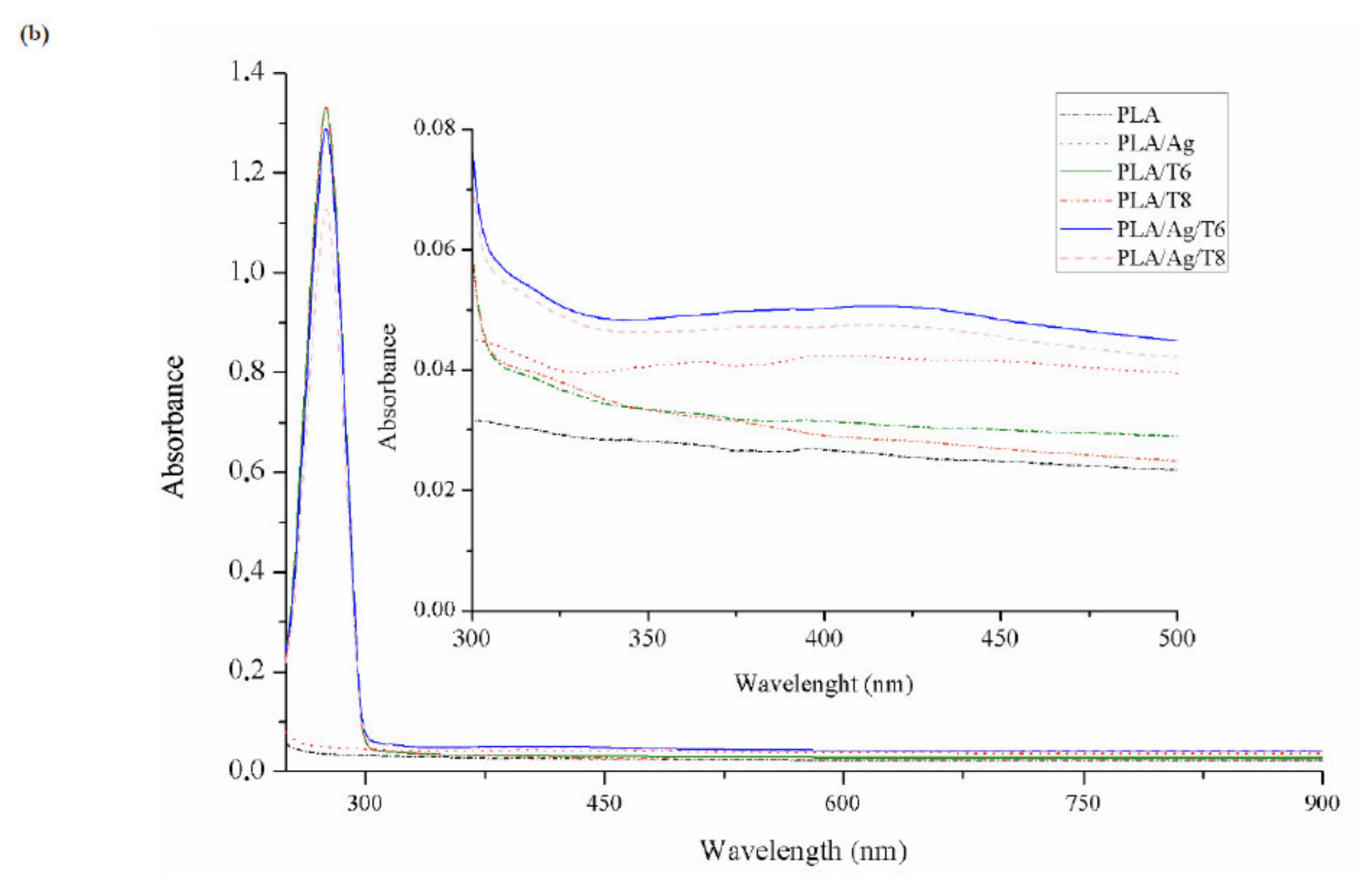
Fourier transform infrared spectroscopy (FTIR) (**a**) and ultraviolet–visible (UV-VI)S (**b**) spectra of neat poly(lactic acid) (PLA) and active PLA-based nanocomposite films.

FTIR and UV-VIS absorption spectra of PLA and PLA active nanocomposite films are shown in Figure 1a,b, respectively. The FTIR spectrum of neat PLA is characterized by several absorption bands, such as those at 770 and 871 cm^−1^, which can be attributed to C-H bond stretching and the intense peak appearing at 1787 cm^−1^ due to the carbonyl group (−C=O) stretching vibration [28]. The FTIR spectra of nanocomposite films confirmed the presence of a significant amount of thymol remaining in the nanocomposite films after processing, since a broad absorption band at 3000–3500 cm^−1^ corresponding to the O-H stretching vibration and the flexion vibration of the methylene group (-CH_2_-) at 806 cm^−1^ (see zoomed area in Figure 1a) was observed for all formulations containing thymol, being a clear indication of the presence of this additive in the processed formulations. The introduction of thymol also resulted in intense absorption bands at 952 cm^−1^ and 1787 cm^−1^, which are indicative of C-O-and –C=O bond vibrations, respectively. These results suggest that thymol reacted with PLA through hydrogen bonding between the phenolic hydroxyl groups of thymol and the carbonyl groups of PLA, as reported by other authors [29,30]. Similar interactions have also been reported between PLA and tea polyphenols [28]. The broad absorption band around 3500 cm^−1^ can also be attributed to the presence of van der Waals interactions between the hydroxyl groups of PLA and the partial positive charge on the Ag-NPs’ surfaces [31].

The presence of thymol in binary and ternary systems after processing was also confirmed by UV-VIS spectrophotometry (Figure 1b). Thymol shows maximum absorption (*λ*_max_) at 274 nm, corresponding to the characteristic band of the π-π* transition, also due to the auxochrome phenolic hydroxyl group present in its structure [32]. Furthermore, those formulations with Ag-NPs showed a low-intensity but characteristic band at around 400 nm, which some authors correlated with the surface plasmon resonance (SPR) transition peak [33,34,35].

**Figure 2:**
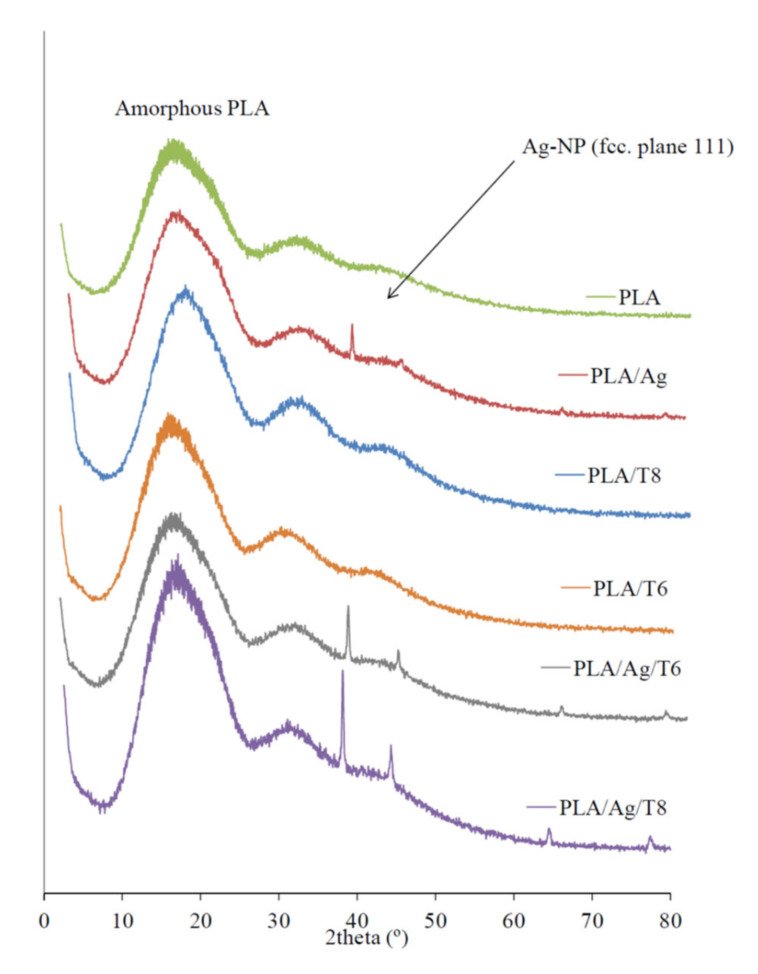
X-ray diffraction (XRD) patterns of PLA and active nanocomposite films.

X-ray diffraction (XRD) patterns of active nanocomposite films with Ag-NPs were clearly indicative of the presence of these nanoparticles embedded into the polymer matrix (Figure 2). While the XRD diffractogram of the mostly amorphous neat PLA only showed the characteristic broad band around 2θ = 20°, the clear and sharp peaks around 38.2° and 44.3° observed in those formulations with Ag-NPs can be attributed to the 111 and 200 crystallographic planes of face-centered cubic (fcc) silver crystals, respectively [34,36].

### 3.2. Quantification of Thymol in PLA-Based Films

The quantification of the final amount of thymol remaining in the active films after processing is essential since the intrinsic high volatility of this additive could result in significant losses during extrusion and further film manufacturing at high temperatures [20]. The final amount of thymol (wt%) was determined in all binary and ternary formulations, with the results shown in Table 1. It was observed that the loss of thymol during extrusion was lower than 30%, regardless of the initial amount present in the formulations (6 or 8 wt%). This result is in accordance with previous studies in our research group [20,37] and it is similar to those reported by other authors. Boonruang et al. developed PLA-based films incorporating thymol and R-(-)-carvone at 10, 15, and 20 wt% by single screw extrusion, and they reported that the remaining concentrations of the volatile compounds in the films were reduced to lower than 50% of the initial loading concentrations [38]. Temperature, residence time of the mixture into the extruder, additive concentration, and the intrinsic volatility of thymol caused by its low melting temperature (50 °C) and vapor pressure (53.33 Pa at 25 °C) were the main factors influencing the permanence in the final blends after processing. Based on the obtained results, it is noticeable that the optimization of the processing conditions allowed thymol to remain inside the extruder for the minimum time to achieve a good dispersion into the polymer matrix, avoiding unnecessary losses.

The PLA/Ag/T8 nanocomposite showed a significantly (*p* < 0.05) higher amount of remaining thymol after processing (around 76%) compared to all other formulations (Table 1). This result may indicate that the loss of thymol during processing could be influenced by the presence of Ag-NPs, which could play a role in retarding the diffusion of thymol molecules through the polymer structure. A similar effect was observed in previous works for active films based on low-density polyethylene (LDPE) or polypropylene (PP) with thymol and different contents of montmorillonite (MMT), with a decrease in the loss of thymol during processing being observed due to the presence of the nanoclay [20,39].

### 3.3. Release Tests from PLA-Based Films

The controlled release of active compounds from a polymer matrix depends on many parameters, such as the molecule mobility, which is determined by the particle size, molecular weight, and geometry of the diffusing compounds [40]. Other important parameters that control the additives’ release are their solubility and diffusivity through the matrix, the pH value, temperature, polymer structure, viscosity, mechanical stress, contact time, and food composition [40,41].

The total amount of silver released from films was directly determined by ICP-MS with LOD and LOQ values of 1.19 µg kg^−1^ and 3.98 µg kg^−1^, respectively. An acceptable level of linearity was obtained from the calibration curve (R^2^ = 0.9972). Results obtained at 2, 4, 6, 12, 24, 48, and 120 h were lower than the LOQ of the method (3.98 µg kg^−1^), indicating a very low Ag release at short times. Table 2 shows the results obtained for the total release of binary (PLA/Ag) and ternary systems (PLA/Ag/T6 and PLA/Ag/T8) after 10 days at 40 °C, expressed as the quantity of silver per kilogram of food simulant after the migration tests. These results were well below the regulatory limits for silver: 0.01 mg Ag kg^−1^ food for “non-authorized substances”, according to commission regulation (EC) No. 450/2009 [42] and specific Ag-NPs migration limits outlined by European Food Safety Authority (EFSA) from food contact materials of 0.05 mg Ag kg^−1^ food [43]. However, there are no current nano-specific migration limits to benchmark the migration of nanoparticles from food contact materials, and these substances should be assessed on a case-by-case basis related to their risk until more information is known about this new technology [42].

Echegoyen et al. described the release of Ag-NPs as a superposition of two simultaneous processes: a surface release and the oxidative dissolution of silver into the ethanol medium [44]. Therefore, the silver species present in ethanol solutions and detected in this study should correspond to Ag^+^ ions liberated by the oxidation of the Ag-NPs [26]. In this sense, it is important to estimate of the eventual migration of Ag-NPs in this type of nanocomposite to reduce human exposure within the safe limits. However, more work with other analytical techniques should be performed to determine the specific migration of silver nanoparticles into food simulants or real foodstuffs. This would allow the evaluation of the toxicological effects of these released nanoparticles and determination of their risk assessment to human health, while maintaining the microbial safety of packaged food [43]. It is important to keep in mind that Ag-NPs are transformed into Ag^+^ when they are in solution, especially in slightly acidic media, meaning the stability of the nanoparticles would be very limited; therefore, a possible toxicological effect should be considered [45]. Furthermore, several studies have demonstrated that Ag-NPs are able to induce cytotoxicity in human cell lines, particularly for those cells with sizes ≤10 nm [46].

Table 2 also shows the amount of thymol released after 10 days of contact between films and ethanol 10% (v/v) at 40 °C. The LOD and LOQ values obtained from the calibration curve were 0.08 mg kg^−1^ and 0.26 mg kg^−1^, respectively. A high level of linearity was obtained from the calibration curve (R^2^ = 0.9999). As expected, the thymol release increased at high contents, showing significant differences between all formulations (*p* < 0.05). The highest migration levels were obtained for the ternary systems, particularly for PLA/Ag/T8 (34.0 ± 1.7 mg kg_simulant_^−1^). This result could give an indication of some protection of Ag-NPs afforded to thymol, preventing losses during processing, which would be in line with previous results obtained for thymol content, shown in Table 1. This effect was also observed by Efrati et al. when blending thymol with different clays, concluding that the increase in the clay content permitted q significant reduction in thymol losses during processing [39].

**Figure 3:**
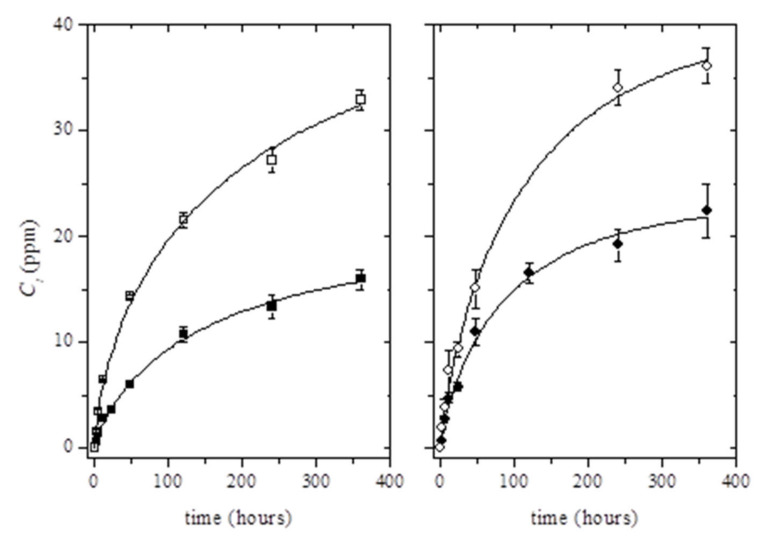
Release kinetics of thymol from binary systems (black dots) and ternary systems (white dots) at 6 wt% (**left**) and 8 wt% (**right**) at 40 °C. Solid lines were obtained by fitting Equation (2) to the experimental data points.

The release kinetics of thymol in ethanol 10% (v/v) was studied for 15 days (Figure 3). As can be seen, the incorporation of Ag-NPs into the polymer matrix resulted in the increase of the total amount of the migrated thymol. Although migration tests were carried out for 15 days, it was observed that the migration steady state was not clearly reached for the whole testing time (Figure 3). Therefore, the estimation of the maximum concentration of thymol able to migrate in ethanol 10% (v/v) at t→∞, *C*_∞_, was performed using the Weibull approach (Equation (2)) (see solid lines in Figure 3):

(2) $$C_{t}=C_{\infty }[1-\exp{\left(-k^{\prime }t\right)}^{d}$$

where *C_t_* is the cumulative concentration (ppm) of thymol (mass of thymol per kilogram of food simulant) released at time *t*, while *k’* and *d* are constant values. Initially, based on empirical observations, Equation (2) can be used as a first assessment of the diffusion mechanism, since *d* and *k’* are closely related to the mechanism and rate release constant, respectively [47,48]. The fit of *C*_∞_ values increased with the incorporation of Ag-NPs into the PLA matrix according to previous results, which was related to the protection of Ag-NPs to thymol losses during processing (Table 3).

The good fitting of values calculated by Equation (2) to experimental data was confirmed by comparing the estimated *C*_∞_ values with the maximum concentration of thymol available to migrate into the food simulant. The computed *C*_∞_ values corresponded to around 19% ± 2% and 22% ± 2% of the initial amount of thymol loaded into PLA matrices (for PLA/T6 and PLA/T8, respectively); and 41 ± 6% and 31% ± 3% of the initial amount of thymol loaded into PLA/Ag/T6 and PLA/Ag/T8, respectively. These results showed that the estimated values were well below the total amount of thymol present in PLA matrices. As a result of the obtained values, it was concluded that the release of thymol could be potentially higher in active nanocomposites than in binary PLA/thymol combinations.

A deeper insight into the mechanism of thymol release was obtained by application of the power law equation, Equation (3), in its logarithm form [49]:

(3) $$\frac{C_{t}}{C_{\infty }}=kt^{n}$$

where *k* and *n* are fitting parameters, giving the later useful information on the release mechanism. The validity of Equation (3) is restricted to *C_t_/C_∞_* < 0.60.

The mean dissolution time (*MDT*), which characterizes the thymol release rate from a given matrix, indicating the thymol-release-retarding efficiency of the polymer can be calculated through Equation (4) [47]:

(4) $$MDT=(\frac{n}{n+1})kt^{n}$$

The analysis of the calculated *n* values (Table 3) showed that the thymol migration from the active nanocomposite films did not follow a diffusion-controlled release (so-called Fickian release), but instead followed a non-Fickian or anomalous release. This effect occurs when the permeant mobility and the polymer segment relaxation rates are similar [50]. It is known that ethanol can act as an aggressive solvent due to structural modifications of the PLA matrix under ethanol sorption, leading to a plasticizer-like effect [51], resulting in molecular rearrangements caused by the increase in mobility of the polymer chain [52]. In this case, it was previously reported that PLA matrices in contact with ethanol 10% (v/v) suffer some molecular modifications, leading to additive release [53].

It is worth noting that the use of Equation (2) in Equation (4) showed that there is a close relationship between the fitting parameter *k’* and (*MDT*)^−1^, following the function (*MDT*)^−1^ = 1.49 (±0.06) *k’* (R^2^ = 0.9929), indicating the validity of both semi-empirical equations used to characterize the migration rate of thymol in PLA-based films. Furthermore, it could be verified that the migration rate calculated taking into account the overall time range was moderately higher than the rate obtained for short-range times, in agreement with the proposed non-Fickian mechanism. However, a more accurate assessment of the migration rate should be carried out on the basis of physical grounds [54]. The release kinetics of thymol from the PLA-based films was evaluated by using the Lagergren first-order and pseudo-second-order rate equations, which can be respectively written in their linear form as:

(5) $$\ln\left(C_{e}-C_{t}\right)=\ln\left(C_{e}\right)-k_{1}t$$

(6) $$\frac{t}{C_{t}}=\frac{1}{k_{2}C_{e}^{2}}+\frac{1}{C_{e}}t$$

where *C*_e_ and *C_t_* are the amounts of thymol migrated into the food simulant (mg L^−1^) at equilibrium and time *t*, respectively; and *k*_1_ and *k*_2_ are the Lagergren pseudo-first-order (s^−1^) and pseudo-second-order rate (L mg^−1^ s^−1^) constants, respectively.

**Figure 4:**
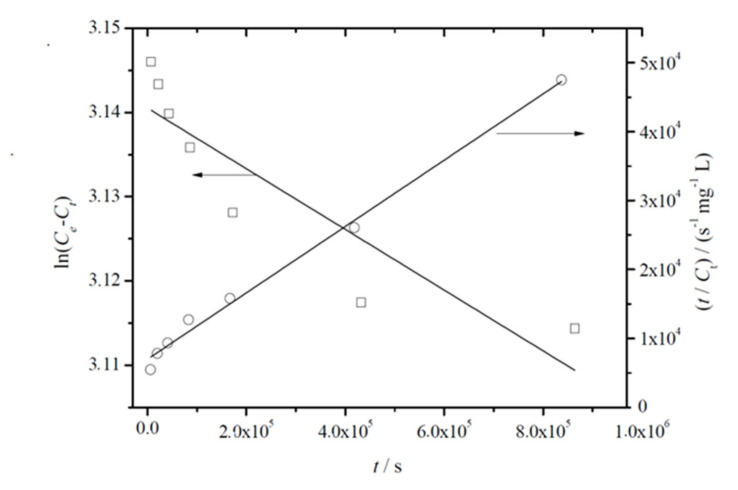
Representative plot of the fitting of linearized forms of pseudo-first (left y*y*-axis, white squares, Equation (5)) and pseudo-second (right y*y*-axis, white dots, Equation (6))-order equations to experimentally released amounts of thymol from PLA/T8 to ethanol 10% (v/v) at 40 °C.

Figure 4 shows the representative plots obtained for the fitting of linearized forms of Equations (5) and (6) to experimental data. From this analysis, joined to the fitting parameters summarized in Table 4, it can be concluded that the pseudo-second-order model showed higher correlation coefficients. It was also observed that *C_e_* values agreed well with *C_∞_*, which was initially estimated and reported in Table 3. Both facts suggest that migration of thymol from PLA-based films follows pseudo-second-order kinetics, which relies on the assumption that physisorption is not the rate-limiting step [55], in agreement with the mechanism described by Equation (3).

On the other hand, in the evaluation of *k*_2_ values obtained for the migration of thymol from active nanocomposite films with Ag-NPs, it was observed that the incorporation of Ag-NPs slowed down the migration process in both formulations. This fact could be due to the interactions between Ag-NPs and thymol molecules with the polymer matrix, where free space could be reduced, producing an increase in molecular mobility and easier interactions between all components.

### 3.4. Antioxidant Activity of Active PLA-Based Films

Considering the significant antioxidant activity of the thymol, the antioxidant properties of this compound released in ethanol 10% (v/v) over 15 days were evaluated using the DPPH method.

**Figure 5:**
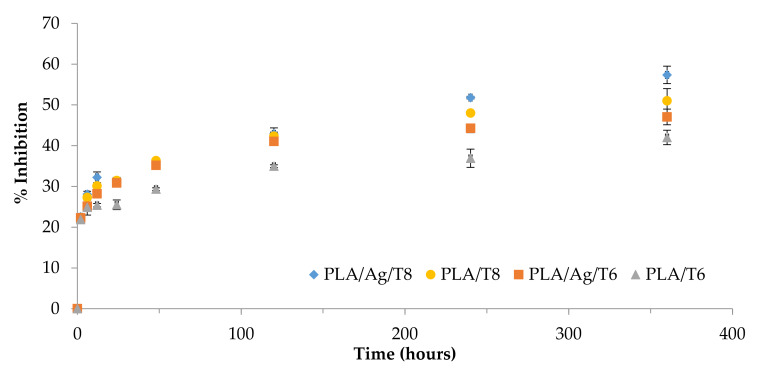
The 2-diphenyl-1-picrilhydrazyl (DPPH) reduction properties of thymol over time.

Figure 5 shows the increase over time of the radical scavenging activity as a result of the controlled release of thymol from the polymer matrix.

Table 2 shows the results obtained for all migration extracts after 10 days in contact with ethanol 10% (v/v). The morphology of the curves could be evaluated by considering a controlled release of thymol in terms of rate. The DPPH radical scavenging ability was significantly higher in migration extracts obtained from ternary systems than those from binary formulations (*p* < 0.05), with the highest value being obtained for PLA/Ag/T8. The antioxidant activity of this formulation compared to that obtained for the binary formulation (PLA/T8) suggests that some interactions between Ag-NPs and thymol could produce an increase in molecular mobility, consequently improving the antioxidant activity of the system, in agreement with previously obtained *k*_2_ values.

A similar dependence between the amount of thymol released and the obtained antioxidant activity was also observed by Ramos et al. in PP-based films using thymol and carvacrol as active additives [56]. These results are also in agreement with previous reports dealing with bioactive compounds, such as thymol obtained from essential oils, herbs, or spices, presenting antioxidant capacity and strong protection of food when released from the polymer matrix [57,58,59,60,61,62].

### 3.5. Antibacterial Activity from PLA-Based Films

The antibacterial activity of neat PLA and nanocomposite films was evaluated to assess their potential in active packaging systems. Gram-negative *Escherichia coli* RB (*E. coli* RB) and Gram-positive *Staphylococcus aureus* 8325-4 (*S. aureus* 8325-4) were selected in this study as representative bacterial strains. The cell viability (%) was evaluated by putting PLA-based films in contact with suspensions of each bacterial strain incubated for 3 and 24 h at 4, 24, and 37 °C, using neat PLA film as control. This response was calculated as the percentage of CFU on PLA film formulations related to CFU of bacteria growth on PLA, set at 100% (Table 5). Adequate temperatures of 4 and 24 °C were selected to evaluate their influence on the antibacterial activity, considering that perishable food is usually kept refrigerated at 4 °C, but under transportation food is more likely to be kept at higher temperatures of storage (24 °C). As expected, neat PLA control film did not show any inhibitory activity against the studied bacteria at the incubation times and tested temperatures.

PLA-based binary and ternary systems showed dose-dependent antibacterial activity for Ag and thymol against *S. aureus* 8325-A and *E. coli* RB strains, however with some differences. A slight reduction in the bacterial growth was observed for binary systems containing thymol against *E. coli* RB, with no significant differences (*p* > 0.05) observed after 3 and 24 h of incubation at 37 °C. Moreover, these films significantly inhibited the growth of *S. aureus* 8325-A after 3 and 24 h, with no significant differences observed at 37 °C after 24 h (*p* > 0.05). These results are in agreement with those obtained in a previous study, where the concentration of thymol added into PP films was not enough to inhibit the growth of *E. coli* RB. The antibacterial activity of thymol has been proposed as consisting of binding to membrane proteins by means of hydrogen bonding, thereby changing the membrane permeability characteristics [63]. Therefore, the antibacterial activity of thymol is strongly dependent on the physico-chemical characteristics and composition of the bacterial membranes [64]. The mechanism of action is based on the disturbance of the cytoplasmic bacterial membrane, disrupting the proton motive force (PMF), electron flow, active transport, and coagulation of cell contents [65]. In fact, most studies investigating the actions of essential oils and their components against food spoilage organisms and foodborne pathogens agree that these compounds (including thymol) are generally more active against Gram-positive than Gram-negative bacteria, such as *E. coli* RB. These bacteria possess an outer layer surrounding the cell wall, which is primarily composed of lipids, proteins, and lipo-polysaccharides, forming a hydrophilic barrier and providing protection against the diffusion of hydrophobic compounds through them. In contrast, the cell wall of Gram-positive bacteria, such as *S. aureus* 8325-A, does not contain lipo-polysaccharides, and consequently thymol can be more susceptible on growth inhibition [37,65].

Active nanocomposite films containing Ag-NPs showed relevant antibacterial activity against both bacteria, with significant differences (*p* < 0.001) at 3 and 24 h compared to the PLA control film. The antibacterial activity of ternary nanocomposites was higher against *S. aureus* 8325-A than against *E. coli* RB, regardless of the incubation time and temperature, confirming similar results reported by other authors [66,67,68]. Erem et al. evaluated the antibacterial activity of PLA fibers with Ag-NPs against *S. aureus* and *Klebsiella pneumonia* (Gram-negative) bacteria, concluding that Ag-NPs were more effective against *S. aureus* [69]. These results have been attributed to the structure and mode of antibacterial action of Ag-NPs, as well as to differences in the cell wall structures of Gram-positive and Gram-negative cells [70,71].

However, the mechanism of action of Ag-NPs has not been well established and several possibilities have been proposed to explain the antibacterial activity of Ag-NPs. Some authors have focused on cell membrane disruption due to the interaction of Ag-NPs with phosphorous- and Sulphur-containing protein compounds, preventing DNA replication. Other studies focused on the binding of the positively charged Ag-NPs with negatively charged bacterial cell membranes, disrupting cell walls and surface proteins [72]. A third mechanism is related to the penetration of Ag-NPs into bacteria, which inactivates enzymes producing H_2_O_2_. All of these possible mechanisms finally lead to the cell death [66]. Furthermore, some studies have also shown that the toxicity of Ag-NPs varies significantly depending on their dimensions and shape, since small nanoparticles have larger relative surface areas for the Ag^+^ release and have higher protein binding efficiencies, allowing them to pass easily through pores in bacterial membranes [73].

The effect of temperature on the antibacterial activity of PLA nanocomposites with thymol and Ag-NPs was also investigated. Results showed the high activity of these ternary systems against both bacteria, with significant differences (*p* < 0.001) obtained for each incubation temperature at 3 and 24 h compared to the PLA control sample, with lower cell viability observed in *S. aureus 8325-4*. The presence of thymol resulted in the films showing an increased antibacterial effect due to the damage caused by both additives to bacterial membranes, permitting their higher crossing, penetration in the cells’ internal parts, and interaction with critical intracellular sites, leading to an improvement in the antibacterial effect.

## 4. Conclusions

The identification of Ag-NPs and thymol in the PLA matrix was successfully carried out, highlighting the presence of both additives in the polymer matrix after processing. Thymol and Ag-NPs were identified and quantified using HPLC-UV and ICP-MS, respectively. These techniques have been shown to be powerful tools for the determination of the migrated compounds based on their release at controlled rates, with thymol release following pseudo-second-order kinetics. The possibility to use PLA as the polymer matrix to host thymol and Ag-NPs has been demonstrated, with potential use as an antioxidant and antibacterial system due to their controlled release. This study has also shown the potential of these PLA-based nanocomposites in incorporating different amounts of thymol with marked antioxidant and antibacterial activity, while Ag-NPs helped to obtain remarkable antibacterial performance. In particular, the ternary formulation PLA/Ag/T8 showed higher positive results concerning antioxidant activity, demonstrating effectiveness in the radical scavenging inhibition using the DPPH method. In conclusion, these results demonstrated that the presence of Ag-NPs and the release of thymol from the PLA matrix resulted in increasing the antibacterial and antioxidant activities of nanocomposites, showing great potential for preservation and shelf-life extension in food packaging applications.

## Figures and Tables

**Table 1:** PLA nano-biocomposites, thickness values, and amounts of thymol quantified in the active films after processing (wt%). Mean ± SD (n = 3).

Formulations	Code	Film Thickness (µm)	Thymol (wt%)
PLA	PLA	35 ± 4 ^a^	n.d.
PLA + Ag 1 wt%	PLA/Ag	39 ± 4 ^a^	n.d.
PLA + thymol 6 wt%	PLA/T6	40 ± 2 ^a^	4.38 ± 0.04 ^a^
PLA + thymol 8 wt%	PLA/T8	41 ± 5 ^a^	5.79 ± 0.07 ^b^
PLA + Ag 1 wt%+ thymol 6 wt%	PLA/Ag/T6	42 ± 3 ^a^	4.41 ± 0.04 ^c^
PLA + Ag 1 wt%+ thymol 8 wt%	PLA/Ag/T8	39 ± 6 ^a^	6.09 ± 0.09 ^d^

Note: n.d.: not detected. Different superscripts within the same column (a, b, c, d) indicate statistically significant different values (*p* < 0.05).

**Table 2:** Thymol and Ag release (ethanol 10% (v/v) after 10 days at 40 °C) and 2-diphenyl-1-picrilhydrazyl (DPPH) scavenging activity (%) of PLA-based films. Mean ± SD (n = 3).

Samples	Thymol and Ag Migrated after 10 Days		DPPH Scavenging Activity (%)
	mg_Thy_ (kg_simulant_)^−1^	µg_Ag-NPs_ (kg_simulant_)^−1^	
PLA	n.d.	n.d.	n.d.
PLA/Ag	n.d.	5.9 ± 0.7 ^a^	n.d.
PLA/T6	13.4 ± 1.1 ^a^	n.d.	36.9 ± 2.2 ^a^
PLA/T8	18.2 ± 2.5 ^b^	n.d.	44.3 ± 1.1 ^b^
PLA/Ag/T6	27.2 ± 0.7 ^c^	7.1 ± 1.8 ^a^	48.0 ± 0.1 ^c^
PLA/Ag/T8	34.0 ± 1.7 ^d^	8.6 ± 0.3 ^a^	51.8 ± 0.3 ^d^

Note: n.d.: not detected. Different superscripts within the same column (a, b, c, d) indicate statistically significant different values (*p* < 0.05).

**Table 3:** Fitting parameters of Equations (2)–(4) to experimental migration data of thymol loaded in binary and ternary systems (ethanol 10% (v/v), 40 °C). Mean ± SD (n = 3).

	PLA/T6	PLA/Ag/T6	PLA/T8	PLA/Ag/T8
Equation (2) Weibull approach				
*C*_∞_ (ppm)	18.7 ± 1.7	42.7 ± 6.4	23.3 ± 1.6	40.6 ± 3.0
*k’* (10^−3^ h^−1^)	5.5 ± 1.3	4.7 ± 2.0	10.4 ± 2.2	8.2 ± 1.9
*d*	0.76 ± 0.05	0.65 ± 0.06	0.77 ± 0.07	0.78 ± 0.06
R^2^	0.9963	0.9965	0.9924	0.9969
Equations (3) and (4). Power law equation				
*n*	0.69 ± 0.03	0.60 ± 0.03	0.63 ± 0.02	0.65 ± 0.04
*MDT* * (h)	104	137	68	84
R^2^	0.9910	0.9869	0.9934	0.9853

* Mean dissolution time (MDT): calculated from Equation (4), taking into account short-range time migration conditions (*C_t_*/*C_∞_* < 0.60).

**Table 4:** Kinetic parameters for migration of thymol from PLA-based films using Equations (5) and (6). Mean ± SD (n = 3).

	PLA/T6	PLA/Ag/T6	PLA/T8	PLA/Ag/T8
Equation (5). First-order rate equation				
*k*_1_ (10^−8^ s^−1^)	3.4 ± 0.4	1.7 ± 0.3	3.6 ± 0.7	2.2 ± 0.3
*C*_e_ (mg L^−1^)	19 ± 1	43 ± 1	23 ± 1	40 ± 1
R^2^	0.8960	0.8986	0.7842	0.9384
Equation (6). Pseudo-second-order rate equation				
*k*_2_ (10^−7^ L mg^−1^ s^−1^)	1.7 ± 0.2	1.2 ± 0.2	3.1 ± 0.3	0.9 ± 0.1
*C*_e_ (mg L^−1^)	19.1 ± 0.1	36.0 ± 2.0	21.4 ± 0.8	44.0 ± 2.0
R^2^	0.9834	0.9824	0.9924	0.9905

**Table 5:** Antibacterial activity of neat PLA and nanocomposite films, expressed as cell viability (%), against *S. aureus 8325-4* and *E. coli RB* strains after 3 and 24 h of incubation at 4, 24, and 37 °C. Mean ± SD (n = 3).

Formulation	*S. aureus* 8325-4		*E. coli* RB	
	3 h	24 h	3 h	24 h
At 4 °C.				
PLA/Ag	51.7 ± 5.7 ^a^	50.4 ± 4.6 ^a^	78.1 ± 6.5 ^a^	65.4 ± 5.4 ^a^
PLA/T6	61.5 ± 5.1 ^a^	87.9 ± 4.2 ^a^	96.6 ± 5.9 ^c^	97.3 ± 4.8 ^c^
PLA/T8	71.9 ± 6.0 ^a^	91.4 ± 4.9 ^b^	92.3 ± 6.8 ^c^	89.6 ± 5.6 ^b^
PLA/Ag/T6	64.2 ± 4.2 ^a^	62.6 ± 3.4 ^a^	71.5 ± 4.7 ^a^	63.9 ± 3.9 ^a^
PLA/Ag/T8	51.3 ± 3.0 ^a^	51.5 ± 2.4 ^a^	69.9 ± 3.4 ^a^	65.9 ± 2.8 ^a^
At 24 °C				
PLA/Ag	50.6 ± 4.7 ^a^	51.5 ± 2.2 ^a^	81.5 ± 4.7 ^a^	72.8 ± 2.7 ^a^
PLA/T6	63.7 ± 4.3 ^a^	88.3 ± 2.0 ^a^	91.5 ± 4.2 ^b^	83.9 ± 2.4 ^a^
PLA/T8	69.5 ± 5.0 ^a^	89.2 ± 2.3 ^a^	89.4 ± 4.9 ^a^	82.6 ± 2.8 ^a^
PLA/Ag/T6	59.3 ± 3.5 ^a^	59.4 ± 1.6 ^a^	69.5 ± 3.4 ^a^	61.4 ± 1.9 ^a^
PLA/Ag/T8	52.5 ± 2.5 ^a^	60.3 ± 1.2 ^a^	59.4 ± 2.4 ^a^	60.2 ± 1.4 ^a^
At 37 °C				
PLA/Ag	53.8 ± 2.4 ^a^	53.6 ± 6.1 ^a^	75.0 ± 3.1 ^a^	69.7 ± 2.7 ^a^
PLA/T6	78.7 ± 5.7 ^b^	91.4 ± 4.3 ^c^	100.0 ± 2.3 ^c^	91.2 ± 3.0 ^c^
PLA/T8	72.0 ± 12.4 ^a^	97.4 ± 14.1 ^c^	96.7 ± 3.6 ^c^	90.4 ± 4.8 ^c^
PLA/Ag/T6	61.4 ± 4.8 ^a^	67.4 ± 1.9 ^a^	72.6 ± 3.3 ^a^	75.8 ± 4.5 ^a^
PLA/Ag/T8	55.6 ± 1.5 ^a^	55.6 ± 0.7 ^a^	77.1 ± 0.9 ^a^	71.7 ± 3.9 ^a^

Data obtained are expressed as percentage of the CFU of bacteria grown on PLA film formulations to CFU of bacteria grown on PLA, set as 100%. Note: *^a^ p* < 0.001; *^b^ p* < 0.05; *^c^ p* > 0.05. For calculation of the *p* values, PLA versus PLA-based nanocomposite film results were compared at 3 and 24 h for *S. aureus 8325-4* and *E. coli RB*.
